# Research Advances on the Role of Lipids in the Life Cycle of Human Coronaviruses

**DOI:** 10.3390/microorganisms12010063

**Published:** 2023-12-28

**Authors:** Cuiling Ding, Yibo Chen, Gen Miao, Zhongtian Qi

**Affiliations:** 1 Department of Microbiology, Faculty of Naval Medicine, Naval Medical University, Shanghai 200433, China; cuilingding@smmu.edu.cn (C.D.); miraclechen03@163.com (Y.C.); 2 Department of Nutrition and Food Hygiene, Faculty of Naval Medicine, Naval Medical University, Shanghai 200433, China; 15021589395@163.com

**Keywords:** coronaviruses, lipids, viral life cycle, antivirals

## Abstract

Coronaviruses (CoVs) are emerging pathogens with a significant potential to cause life-threatening harm to human health. Since the beginning of the 21st century, three highly pathogenic and transmissible human CoVs have emerged, triggering epidemics and posing major threats to global public health. CoVs are enveloped viruses encased in a lipid bilayer. As fundamental components of cells, lipids can play an integral role in many physiological processes, which have been reported to play important roles in the life cycle of CoVs, including viral entry, uncoating, replication, assembly, and release. Therefore, research on the role of lipids in the CoV life cycle can provide a basis for a better understanding of the infection mechanism of CoVs and provide lipid targets for the development of new antiviral strategies. In this review, research advances on the role of lipids in different stages of viral infection and the possible targets of lipids that interfere with the viral life cycle are discussed.

## 1. Introduction

Coronaviruses (CoVs) are emerging pathogens with serious life-threatening effects on human health. Seven known CoVs can infect humans: human coronavirus 229E (HCoV-229E), HCoV-OC43, HCoV-NL63, HCoV-HKU1, severe acute respiratory syndrome coronavirus (SARS-CoV), Middle East respiratory syndrome coronavirus (MERS-CoV), and SARS-CoV-2 [1]. At the beginning of the 21st century, SARS-CoV and MERS-CoV have brought great burden to the economy and health of some countries, and have become one of the important global public health issues [1]. The emergence of SARS-CoV-2 at the end of 2019 triggered a large-scale worldwide viral pneumonia pandemic, which was historically rare [2,3]. To date, there are no specific drugs for CoVs, except for several drugs approved based on emergency authorization for SARS-CoV-2 infection. Moreover, the constant emergence of new SARS-CoV-2 mutations presents a major long-term challenge for antiviral therapeutics.

Lipids are essential nutrients that play important roles in several fundamental cellular and physiological functions, including membrane permeability, cell signaling, cell death, and survival pathways [4]. Moreover, as important components of cell and viral membrane structures, lipids may also play a central role in viral infection [5], including cholesterol, glycosphingolipids, fatty acids (FAs), and linoleic acid (LA). During viral invasion, lipid rafts provide receptors or cofactors for all viral types to facilitate viral entry and membrane fusion [6,7]. Lipids, which are involved in membrane fusion, encapsulation, and transformation, promote viral replication [5]. However, lipid metabolism in host cells can be altered by viral infections [8]. Host lipid remodeling is significantly associated with pathogenic CoV transmission; therefore, lipid regulation can be used as a target for CoV-specific drugs [9].

During CoV infection, lipids have been shown to play important roles in multiple processes, including viral entry, membrane fusion, biosynthesis, assembly, and release. Katia et al. summarized the interaction of CoVs with lipid rafts and autophagy, but focused only on fat rafts [10]. Jun et al. described in detail the link between CoV infection and cholesterol metabolism [11], while Ulrich et al. summarized the interaction between SARS-CoV-2 infection and lipids [12]. Philippe et al. summarized the role of double-membrane vesicles (DMVs) in SARS-CoV-2 and other positive-sense single-stranded RNA viruses [13]. However, there is currently no comprehensive summary of the role of lipids in the human coronavirus life cycle. In this review, research advances in the role of lipids in different stages of human CoV infection and the possible targets of lipids that interfere with the human CoV life cycle are discussed. A better understanding of the roles of lipids during CoV infection is required for the exploration of viral pathogenesis, and a basis will be provided for the development of anti-CoV treatment strategies.

## 2. Lipids Affect the CoV Life Cycle

### 2.1. The Role of Lipids in CoV Entry

CoVs can undergo membrane fusion directly on the cell surface after binding to specific receptors, or in the endosomal compartment of the cell after internalization by endocytosis [14,15], which requires the help of lipids [16,17]. Lipids play a key role in the initial stages of virus–host cell membrane interactions, the first step of which is viral adhesion [18].

Lipid rafts are microdomains on cell membranes enriched in cholesterol, glycosphingolipids, and proteins [19,20,21,22,23]. The internalization of pathogens is always mediated by lipid rafts [24,25], which can help CoV enter host cells, as well as their genome release [26,27], and provide a platform for receptors in the human body involved in physiological activities, including cell signaling, synaptic activity, immune response, membrane trafficking, and cytoskeletal reconstruction [19,28,29,30,31]. By interacting with lipid rafts, the virus regulates the normal life processes of the host cell and enters the host cell [32,33].

First, both receptors and cofactors concentrated in lipid rafts affect the fusion of the virus with the host cell membrane, which assists with viral entry into the host cell [34,35,36,37]. Lipid rafts provide a platform for the receptors of CoVs, including angiotensin-converting enzyme-2 [38,39,40,41] (ACE2, the receptors for HCoV-NL63 [42], SARS-CoV [41], and SARS-CoV-2 [43]), dipeptidyl peptidase 4 [44] (DPP4, the receptor for MERS-CoV [45]), and aminopeptidase [46] (APN, the receptor for HCoV-229E [47]). In addition, lipid rafts, which are required for mouse hepatitis virus (MHV) entry and membrane fusion [48], act as attachment factors to promote the absorption of the infectious bronchitis virus (IBV) absorption before entering the cell [39]. Moreover, viruses can enter host cells via caveolin or clathrin-mediated membrane fusion or endocytosis on lipid rafts [49].

Second, the integrity of the lipid rafts is associated with viral entry. Cryoelectron microscopy was used to examine the structure of the spike (S) protein in SARS-CoV, MERS-CoV, and SARS-CoV-2, and three binding sites were identified in the receptor-binding domain (RBD) that are firmly bound to LA [50]. This suggests that the integrity of lipid rafts can regulate the interaction between the receptors on host cells and the SARS-CoV-2 viral S protein, thereby affecting viral entry [51]. One study demonstrated that methyl-β-cyclodextrin (MβCD) can promote cholesterol depletion, directly affecting ACE2 receptors and transmembrane protease serine 2 (TMPRSS2) on the lipid rafts, resulting in alterations in the structural integrity of the lipid rafts, thereby inhibiting the entry of SARS-CoV-2 into cells [52]. The integrity of lipid rafts has also been reported to be required for productive infection of pseudotyped SARS-CoV [40].

Third, cell membrane lipid rearrangements facilitate CoV entry. The life cycles of positive-stranded RNA viruses are closely associated with rearranged intracellular membranes [53]. The structural rearrangements of the S protein of SARS-CoV-2 can affect the membrane fusion process between the virus and the host cell, thereby facilitating viral entry [54]. One study revealed that bazedoxifene acetate can alter the distribution of cholesterol on the membrane and endosomal acidification, affecting the entry of SARS-CoV-2 into host cells and exerting antiviral effects [55]. Lipid rafts permit membrane rearrangement to facilitate the entry of transmissible gastroenteritis viruses (TGEVs) into host cells [56].

Fourth, the lipid metabolism also affects CoV entry. Cholesterol metabolism is a key host pathway that promotes CoV infections, including those of SARS-CoV-2, HCoV-229E, and HCoV-OC43 [57], whereas cholesterol dysregulation reduces viral entry [57,58,59,60]. CoVs enter cells either through fusion or endocytosis in a cholesterol-dependent manner [61]; therefore, the cholesterol metabolism exerts an important influence on the viral entry stage [62]. Efficient removal of cholesterol from the membrane microdomain disrupts the lipid raft-regulated signaling pathway, eliminates lipid raft-associated proteins [10,34,63,64], and alters the activities of the receptors ACE2 and TMPRSS2 on the lipid rafts, which in turn interferes with the viral internalization process [65,66,67].

**Figure 1:**
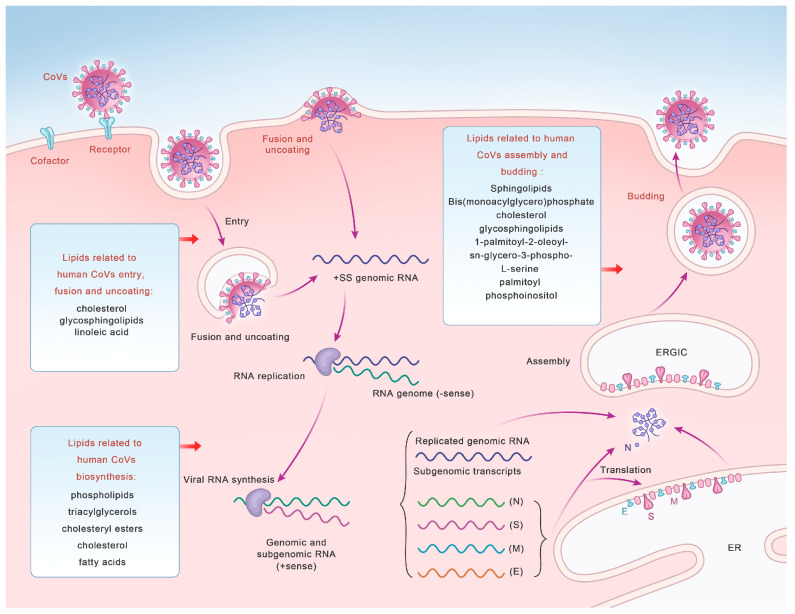
A diagram illustrating the life cycle of coronaviruses and lipids related to human CoV entry, fusion and uncoating, synthesis, and assembly and budding.

In conclusion, lipid rafts can provide a platform for the receptors and cofactors of CoVs, and their integrity is related to viral invasion. Lipid membrane rearrangement and metabolism also affect viral invasion. These results indicate that lipids are essential for the life cycle of CoVs and provide a basis for the subsequent use of regulatory lipids for anti-CoV entry (Figure 1, Table 1).

### 2.2. The Role of Lipids in CoV Membrane Fusion

The mature CoV virions are surrounded by a lipid bilayer which contains various glycoproteins, the most important of which is the S protein, which is responsible for target cell binding and membrane fusion. The S1 subunit of S proteins is responsible for binding to the cellular membrane surface receptors. The S2 subunit is essential for bridging the viral and host cell membranes. After S glycoproteins are cleaved by furin protease, the S2 subunit exposes and anchors the membrane and undergoes conformational changes and creates a fusion pore, thereby promoting the membrane fusion [68]. During SARS-CoV-2 infection, S proteins are lipid-modified through the sequential action of the S-acyltransferase ZDHHC20. Moreover, acylation of the S generates cholesterol-rich lipid domains within viral envelopes, which allows the formation of viruses with enhanced fusion capacity [69]. One study screened 3000 approved drugs to search for inhibitors of S-driven syncytia in SARS-CoV-2 [70]. One of the most effective drugs can suppress the activity of TMEM16F, which can transport the phosphatidylserine (PS) from the cytofacial leaflet to the exofacial leaflet of the cell membrane, therefore inhibiting the virus–cell membrane fusion and syncytia formation [71]. The downregulation of TMEM16F also decreases the syncytia formation. However, TMEM16F has no function in MERS-CoV S protein-mediated syncytia formation [70]. Briefly, lipids play distinct roles in the fusion of multiple CoVs (Figure 1, Table 1).

### 2.3. The Role of Lipids in CoV Biosynthesis

Viral replication processes can be controlled by host lipogenesis pathways, which aid in forming viral replication complexes, enable optimal function, and produce the energy required for viral replication [72,73,74].

One study revealed that SARS-CoV-2 infection can alter the lipid metabolism in host cells, making the internal environment conducive to viral assembly and replication [75]. Cholesterol and FA are the main components of the viral membrane, for which the metabolism is closely related to the replication of various viruses [76]. In addition, intracellular cholesterol biosynthesis and transport systems are related to viral replication [77,78,79].

The expression and activity of key enzymes involved in lipid biosynthesis can be enhanced by viruses to facilitate their replication processes smoothly (Figure 1, Table 1). Both SARS-CoV-2 and MERS-CoV contribute to the production of lipid anabolic enzymes, including fatty acid synthase (FASN) and acetyl-CoA carboxylase (ACC1) [80,81]. FASN is a key cellular enzyme in the intracellular palmitate synthesis process [82]. FASN promotes palmitate synthesis, which can be used as a raw material for other lipid syntheses, thereby promoting the construction of viral envelopes and replication organelles [83]. Some enveloped viruses, including hepatitis B virus [84], dengue virus [85,86], hepatitis C virus [87], and West Nile virus [88,89], upregulate the expression and enhance FASN activity, whereas the knockout of FASN significantly impairs SARS-CoV-2 replication [83,90], while exogenous FA supplementation can rescue this damage [91]. The antiviral effects of cholesterol and its metabolizing enzymes or corresponding natural products are closely associated with various steps in CoV replication [11]. Cholesterol is essential for the pathological syncytia formation of SARS-CoV-2 and is thought to contribute to the replication and evasion of host immune responses [51], whereas 3-hydroxy-3-methyglutaryl CoA reductase (HMGCR) is a rate-limiting enzyme for cholesterol synthesis [92] that can affect viral replication by regulating cholesterol levels [11].

Lipids can facilitate viral replication by providing specific signals for the function and localization of viral proteins (Figure 1). SARS-CoV-2 can use the host metabolic pathways to induce major cellular lipid rearrangements, which in turn facilitate viral replication [93,94]. These interactions can lead to the rearrangement of host intracellular membranes to form DMVs that contain cholesterol, FA [95,96], and viral dsRNA [97], which can anchor viral replication transcription complexes [98] and serve as efficient replication and assembly sites for CoV genetic materials [53,99,100]. Following CoV infection, the formation of organelle-like replicative structures consisting of DMVs and convoluted membranes can lead the localization of those nonstructural proteins (NSPs) involved in RNA synthesis [101,102]. The NSPs of CoVs can induce the rearrangement of lipid rafts on cell membranes and the formation of DMVs in the cytoplasm. Sterol regulatory-element-binding proteins (SREBPs) on the endoplasmic reticulum (ER) can bind to promoters of genes involved in lipid biosynthesis to regulate the lipid metabolism and homeostasis in host cells [103]. SREBPs are highly upregulated in patients infected with MERS-CoV and influenza A virus, and can affect downstream viral protein palmitation and DMV formation in the host [104]. As an important lipid-processing enzyme, cytoplasmic phospholipase A2α enzyme (CPLA2α) is also essential for DMV formation and CoV replication [102]. Additionally, cPLA2α asymmetrically cleaves phospholipids in a lipid bilayer, which induces membrane curvature and may increase the formation of vesicle membrane structures, including lipid droplets (LDs) [102].

LDs are composed of triacylglycerols (TAGs), unsaturated and saturated chains, and cholesteryl esters (CEs) [105]. As lipid-rich organelles that regulate the lipid homeostasis and metabolism [106,107], LDs play an important role in the replication of single-stranded RNA viruses, including CoVs [97,108]. One study demonstrated that SARS-CoV-2 induced the reprogramming of the lipid metabolism in host cells, which can favor viral replication by the accumulation of LDs [109]. LDs bear a high degree of similarity to the SARS-CoV-2 proteins, whose increased levels can significantly contribute to viral replication [109]. SARS-CoV-2 infection directly affects the host cell lipid metabolism and synthesis of LDs, suggesting that the virus may exploit host metabolism and use LDs as a replication platform to favor its replication [109]. MERS-CoV infection leads the accumulation of LDs and cholesterol by triggering the SREBP pathway, while SREBP1 and 2 are essential for viral replication [97].

### 2.4. The Role of Lipids in CoV Assembly and Budding

Lipids also play an integral role in CoV assembly and budding (Figure 1, Table 1). Several studies have shown that sphingolipids mediate signal transduction, interactions with internal membranes, the lipid metabolism during replication, and viral assembly and budding [110]. Bis(monoacylglycero)phosphate (BMP), a specific lipid of late endosomes which is involved in vesicular transport and pH-dependent vesicle budding [111], is associated with lysosomal stability, function, enzyme activation, and endosomal trafficking, and can affect membrane curvature and protein cofactor recruitment [112], thus affecting viral budding.

Specific lipid microdomains, such as lipid rafts and LDs, can also play critical roles in viral morphogenesis and budding. Lipid microdomains contribute to the introduction and concentration of viral components at the budding site [113]. During SARS-CoV-2 infection, S proteins undergo lipid modification via S-acyltransferases, which control S biogenesis and degradation, and affect the formation of lipid domains rich in cholesterol and sphingolipid in the early Golgi apparatus, where viral budding occurs [69]. Electron microscopy analysis of Vero cell infection with SARS-CoV-2 showed that viral particles colocalized with LDs, suggesting that LDs may serve as an assembly platform for CoVs [109].

Envelope (E) and membrane (M) proteins are closely related to the assembly and release processes of CoVs, in which lipids also play an important role. Lipid–protein contact analyses have demonstrated that M protein dimers preferentially associate with 1-palmitoyl-2-oleoyl-sn-glycero-3-phospho-L-serine (POPS) and palmitoyl phosphoinositol (POPI) lipids, suggesting that M proteins dynamically rearrange the ER–Golgi intermediate compartment (ERGIC) membrane to make the intracellular lipid environment suitable for viral release. Not only are POPI lipids able to promote membrane curvature [114,115], they also have a high affinity for M protein dimers, which can induce the cell membrane to curve to a state conducive to viral release [116]. Monte Carlo simulations showed that peptide–lipid interactions can yield pentamer clusters, which is likely important for E-mediated viral assembly and budding [117].

In summary, a suitable lipid environment is crucial for the successful completion of viral assembly and budding (Figure 1).

**Table 1:** A table summarizing the lipids involved in the life cycle of human coronaviruses.

CoV Life Cycle	Lipid	Function	References
entry	Lipid rafts	platform and docking site for CoV entry and genome release	[24,25,26,27]
	Cholesterol	component of lipid rafts	[19,20,21,22,23]
	Glycosphingolipids	component of lipid rafts	[19,20,21,22,23]
	Linoleic acid	binding to the receptor-binding domain of CoVs	[50]
biosynthesis	Double-membrane vesicles	efficient replication and assembly sites for CoV genomic RNA	[53,99,100]
	Lipid droplets	regulating lipid metabolism to favor replication	[97,106,107,108]
	Cholesterol	associating with various steps in CoV replication	[77,78,79]
	Fatty acids	influencing the production of replication organelles	[83,90,91]
assembly and budding	Sphingolipids	mediating viral assembly and budding	[110]
	Bis(monoacylglycero)phosphate	affecting membrane curvature and protein cofactor recruitment	[111,112]
	Lipid rafts and lipid droplets	contributing to the introduction and concentration of viral components at the budding site	[113]
	1-palmitoyl-2-oleoyl-sn-glycero-3-phospho-L-serine and palmitoyl phosphoinositol	facilitating the membrane curvature	[114,115]

## 3. Anti-CoV Use of Lipids

As mentioned above, CoV infection can be influenced by lipids, which have been suggested as new targets for anti-CoV drugs and treatments [8,118,119,120,121,122,123,124,125].

### 3.1. Antivirals Target CoV-Entry-Related Lipids

**Figure 2:**
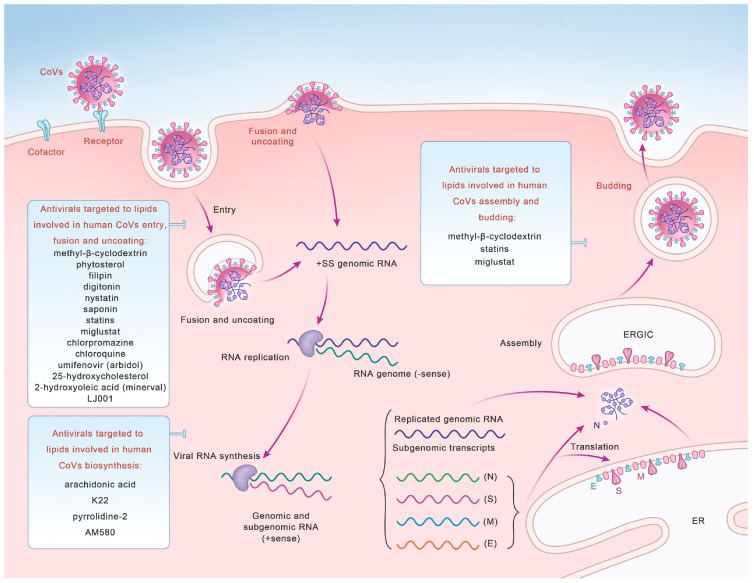
Potential antivirals targeted to lipids that can be used as broad-spectrum antiviral drugs to inhibit human CoV entry, fusion and uncoating, synthesis, and assembly and budding.

Cholesterol-rich lipid rafts are crucial components of the viral envelope [126] and play indispensable roles in the entry of CoVs. Therefore, drugs targeting lipid rafts are beneficial for inhibiting CoV entry and exerting antiviral effects [127,128] (Figure 2, Table 2).

Cholesterol is responsible for the stability of lipid rafts; once damaged, it affects the integrity of the lipid raft, thereby inhibiting CoV entry. MβCD can deplete cholesterol in the host, which lead to the destruction of lipid rafts and impaired viral envelope integrity, thereby reducing the infectivity of influenza viruses, IBV, TGEV, and SARS-CoV [39,129,130]. Disruption of cholesterol on lipid rafts can prevent the transfer of ACE2 to the cell surface, thereby significantly inhibiting the binding of SARS-CoV-2 to its receptors [131]. Researchers have revealed that cholesterol depletion by MβCD can significantly reduce ACE2 binding to the viral S protein, and that the dose of ACE2 expression is positively correlated with SARS-CoV replication [132]. Decreased cholesterol levels and altered ACE2 and TMPRSS2 activities on lipid rafts can inhibit the viral internalization of SARS-CoV-2 [65,66,67]. Similarly, as lipophilic molecules, phytosterols can interact with lipid rafts, reduce cholesterol, and alter the stability of membrane structures, thereby exerting antiviral effects [8]. Other molecules, including filipin, digitonin, nystatin, and saponin, disrupt lipid rafts within a short period by directly removing cholesterol [133], which may also exert good antiviral efficacy [134].

Membrane cholesterol content and ATP-binding cassette transporter A1 (ABCA1) expression have been shown to be closely related to susceptibility to viral infection. The stimulation of ABCA1 expression with liver X receptor (LXR) agonists can inhibit CoV entry by inhibiting the physiological pathways responsible for cholesterol efflux [64,132].

Statins and miglustat, inhibitors of cholesterol and sphingolipid biosynthesis, can reduce the supply of cholesterol and sphingolipids and influence the composition of lipid rafts [131]. Statins can exert antiviral effects by inhibiting the entry or replication stage of SARS-CoV-2 [135]. Other direct inhibitors of endocytosis include chlorpromazine, chloroquine, and umifenovir (arbidol) [18,136,137].

BMP is a structural isomer of phosphatidylglycerol that controls the distribution of cholesterol in cells and regulates the production of oxidized sterols, such as 25-hydroxycholesterol (25-HC) [138]. As a potent inhibitor of SARS-CoV-2 infection, 25-HC limits the viral membrane fusion process by blocking the release of cholesterol in late endosomes [127]. Thus, BMP can affect viral infections [139].

The emerging field of membrane lipid therapy (MLT) has flourished [140], aiming to modulate lipids in biofilms in patients [141] by targeting the cell membrane itself instead of specific proteins on the cell membrane. The disturbance of host cell membranes caused by CoV infection can also be alleviated by treatments targeting the cell membranes. 2-Hydroxyoleic acid (minerval) is considered to be a CoV membrane-targeted drug, which can interact with membrane lipids and change the composition and structure of host cell membranes, thereby affecting CoV entry [142].

As a membrane-binding compound [143,144], LJ001 can exert specific antiviral effects at notably low doses, which means it can selectively affect viral membranes while the host cell membranes remain stable [143,144,145]. LJ001 targets unsaturated phospholipids and leads to the increased hydroxylation of unsaturated fatty acids, affecting the intrinsic structure of the lipid bilayer and thus the viral membrane properties [145].

### 3.2. Antivirals Target CoV-Biosynthesis-Related Lipids

Lipids affecting CoV biosynthesis also exert significant antiviral effects (Figure 2, Table 2). The lipid profiling of hCoV-229E-infected cells revealed that 24 lipids, primarily lysophospholipids and FA, were significantly upregulated in infected host cells [9]. Both LA and arachidonic acid (AA), together with their metabolites, play multiple roles in the mechanisms of CoV infection and the host immune response [146]. The results showed that exogenous supplementation of LA and AA in infected host cells may interfere with the LA–AA metabolic axis, thereby significantly inhibiting the replication of HCoV-229E [147].

DMVs play vital roles in the process of CoV replication, so drugs targeting DMVs can significantly inhibit viral replication, thereby exerting antiviral effects. A compound called K22 inhibits the formation of DMVs at an early stage of the viral life cycle, affecting viral genome replication and transcription, which in turn prevents viral infections [148]. It inhibits the formation of replication–transcription complexes (RTCs) by interacting with nsp6 in DMV, which in turn affects both animal and human CoV (including HCoV-229E, feline coronavirus, IBV, MERS-CoV, MHV, and SARS-CoV) replication [149]. The retinoid receptor α (RAR-A) agonist AM580 can exert its antiviral activity by binding to SREBP in host cells, which significantly inhibit DMV production and interrupt the life cycle of MERS-CoV and influenza A viruses [104]. Moreover, cPLA2α also plays an important role in DMV formation. The use of pyrrolidine-2 (Py-2), a highly specific inhibitor of cPLA2α, on HCoV-229E-infected cells can significantly inhibit the formation of DMV and its related RTCs, thus exerting antiviral effects [102].

Targeting the key lipid synthesis enzymes that affect CoV replication can also inhibit viral infection. As an important enzyme in lipid metabolism, AMP-activated protein kinase (AMPK) can directly inhibit lipid synthesis by inducing phosphorylation of the key enzyme ACC1, and can also disrupt normal lipid metabolism and homeostasis by affecting SREBP-1 [83]. AMPK can reduce lipid biosynthesis and the formation of CoV replication organelles, thus inhibiting virus production and exerting antiviral effects [83].

### 3.3. Lipid Therapy in SARS-CoV-2 Infection

Growing evidence suggests that lipid therapy can be used as a strategy to treat COVID-19 [118,119,121,122,123]. The increase in AA in patients with severe COVID-19 can promote the production of cytokines and lead to cytokine storms [121,150], which induce the release of unsaturated FA in patients, constituting a defense mechanism against viruses [50]. Some therapeutic options use AA and other polyunsaturated FAs together with their derivatives as antiviral molecules to influence cell membrane fluidity, regulate ACE2 receptors, suppress inflammation, enhance healing, and augment the phagocytosis of macrophages and other immunocytes, thus inactivating encapsulated viruses and inhibiting their proliferation in host cells [118,119,121,122,123]. Furthermore, direct binding between FA and SARS-CoV-2 proteins may affect SARS-CoV-2 pathogenicity, suggesting that FA can be considered as a strategy for antiviral activity [151].

Plasmalogens promote the formation of host cubic membranes (CMs) to support the body in various homeostatic conditions, including CoVs infection [152]. Dysregulated plasmalogen levels in patients infected with CoVs suggest that plasmalogen can be considered as an antiviral prophylactic and lipid biomarker for CoV infection [152]. Sphingolipids can regulate host cell membrane properties, including viscosity and tension, making them promising new targets for disease therapeutic interventions [153,154,155]. In addition, sphingolipid expression levels in humans are regulated in numerous diseases, including significantly elevated levels of ceramide and glucosylceramide, which are often found in infected cells [153,154,155,156,157,158]. Therefore, targeting sphingolipids can be considered an effective lipid treatment regimen against complications related to SARS-CoV-2 infection [159].

**Table 2:** A table summarizing potential antivirals targeted to lipids to inhibit the human coronavirus and exert broad-spectrum antiviral effects.

CoV Life Cycle	Lipid Inhibitors	Function	References
entry	methyl-β-cyclodextrin	depleting cholesterol to impair viral envelope integrity	[39,129,130]
	phytosterol	reducing membrane cholesterol and destabilizing the membrane structure	[8]
	filipin, digitonin, nystatin, and saponin	disrupting lipid rafts within a short period by directly removing cholesterol	[133]
	statins and miglustat	inhibitors of cholesterol and sphingolipid biosynthesis	[131,135]
	chlorpromazine, chloroquine, and umifenovir (arbidol)	direct inhibitors of endocytosis	[18,136,137]
	25-hydroxycholesterol	blocking the release of cholesterol in late endosomes	[127]
	2-Hydroxyoleic acid (minerval)	interacting with membrane lipids and altering the composition and structure of host cell membranes	[142]
	LJ001	selectively impacting viral membranes	[143,144,145]
biosynthesis	arachidonic acid	interfering with the LA–AA metabolic axis to significantly inhibit the replication	[147]
	K22	inhibiting DMV formation	[149]
	pyrrolidine-2	inhibitor of cytosolic phospholipase A2α	[102]
	AM580	binding to SREBP	[104]
assembly and budding	methyl-β-cyclodextrin	depleting cholesterol to impair viral envelope integrity	[39,129,130]
	statins and miglustat	inhibitors of cholesterol and sphingolipid biosynthesis	[131,135]

## 4. Conclusions

At the beginning of the 21st century, CoVs, such as SARS-CoV, MERS-CoV, and SARS-CoV-2, have become endemic to different parts of the world, causing severe acute respiratory lesions that can cause a large number of deaths in a short period of time, placing a heavy burden on the global health system. Lipids are important components of cell and viral membranes and are involved in several cellular physiological functions. In addition, lipids affect the viral life cycle, including viral invasion, membrane fusion, biosynthesis, and assembly. The important role played by lipids in the life cycle of CoVs provides a novel strategy that differs from traditional antiviral strategies. Since lipids affect multiple processes of viral infection and are less affected by viral mutations, treatments targeting host lipids will make the antiviral effect more comprehensive and effectively address the problem of immune escape caused by high viral variability. Based on the close relationship between lipids and viral infections, numerous anti-CoV lipids have been discovered, including MβCD, statins, BMP, minerval, LA, AA, FA, and sphingolipids.

In summary, lipids and viral infection can be described as mutually influencing each other, and seeking the best antiviral strategy without affecting the human metabolism will be a primary focus of future research.

## Data Availability

Data available on request from the authors.
